# *Lactobacillus delbrueckii* subsp. *allosunkii* and *lactis* as emerging human uropathogens in elderly patients

**DOI:** 10.1128/jcm.02072-24

**Published:** 2025-04-23

**Authors:** François Guérin, Mohamed Sassi, Francois Gravey, Asma Zouari, Benjamin Quenet, Maxime Lecourt, Pauline Ract, Charlotte Michaux, Michel Auzou, Christophe Isnard, Vincent Cattoir

**Affiliations:** 1 INSERM UMR 1230 BRM, Université de Rennes 27079, Rennes, France; 2 CHU Rennes, Service de Bactériologie-Hygiène Hospitalière, Rennes, France; 3 CHU Rennes, CNR de la Résistance aux Antibiotiques (laboratoire associé ‘Entérocoques’), Rennes, France; 4 CHU de Caen, Service de Microbiologie, Caen, France; 5 INSERM UMR 1311 DYNAMICURE, Normandie Université 357634, Caen, France; Johns Hopkins University, Baltimore, Maryland, USA

**Keywords:** lactobacilli, *Lactobacillus delbrueckii*, subspecies, UTI, urine

## Abstract

*Lactobacillus delbrueckii* has been considered a very rare cause of human urinary tract infections (UTIs). However, little is known about its clinical significance and antimicrobial susceptibility, and genomic data from clinical isolates are lacking. This study aimed at analyzing clinical, microbiological, and genomic data of *L. delbrueckii* urinary isolates. All *L. delbrueckii* isolates collected from patients hospitalized in a French university hospital from 2014 to 2016 were included. Clinical and biological data were gathered. Species identification was performed by matrix-assisted laser desorption/ionization time-of-flight (MALDI-TOF) mass spectrometry, and MICs were determined using the broth microdilution method. Whole genome sequencing (WGS) was conducted (Illumina MiSeq 2 × 300 bp), and genomes were compared using three approaches (multilocus sequence typing [MLST], average nucleotide identity [ANI], and core genome single nucleotide polymorphism [SNP]). From 2014 to 2016, 48 isolates of *L. delbrueckii* were recovered from the urine of 48 patients (mean age = 84 years; sex ratio M/F = 0.04). Nearly half (44%) of patients were diagnosed with a UTI, and all had significant cultures (≥10^5^ CFU/mL) with a positive direct examination in >90% of cases. The majority of isolates were susceptible to most antibiotics (especially β-lactams), whereas they seemed intrinsically resistant to fosfomycin and metronidazole. Subspecies identification was consistent across the three approaches, showing that most *L. delbrueckii* isolates belonged to subspecies *allosunkii* (*n* = 40; 83%), followed by subspecies *lactis* (*n* = 8; 17%). Two isolates were resistant to tetracycline (MIC >16 mg/L) and both harbored the *tet*(W) gene. This study demonstrates the uropathogenic role of *L. delbruekii* subspecies *allosunkii* and *lactis*, particularly in elderly female patients.

**IMPORTANCE:**

This largest case series of urinary tract infections (UTIs) caused by *Lactobacillus delbrueckii* clearly demonstrates the uropathogenic role of this species (especially the subspecies *allosunkii*) in human UTIs, particularly in elderly female patients and those with underlying comorbidities. This study may change practice in two ways: (i) clinical laboratories, which typically consider lactobacilli from urine samples as contaminants, may need to reassess this practice; (ii) patient care can be improved by prescribing appropriate antibiotics for these underdiagnosed UTIs. *L. delbrueckii* should be considered an actual pathogen when it is significantly found in the urine of predisposed patients with clinical and/or biological signs of infection. While matrix-assisted laser desorption/ionization time-of-flight (MALDI-TOF) mass spectrometry allows reliable identification of *L. delbrueckii*, there is also a need for better discrimination between subspecies (especially *allosunkii* and *lactis*). Since *L. delbrueckii* isolates are usually susceptible to many antibiotics, we recommend β-lactams (especially aminopenicillins) for the treatment of those UTIs.

## INTRODUCTION

Urinary tract infections (UTIs) are among the most common human infections in both community and hospital settings, affecting 150 million people each year worldwide, with significant morbidity and high medical costs (1). In both settings, *Enterobacterales* are responsible for the vast majority of UTIs, predominantly uropathogenic *Escherichia coli* isolates (1). Besides Gram-negative bacilli, UTIs can also be caused by Gram-positive bacteria such as enterococci, *Staphylococcus saprophyticus*, or *Streptococcus agalactiae* (2). Furthermore, emerging Gram-positive species have recently been described as true uropathogens: *Actinotignum* (formerly *Actinobaculum*) *schaalii* and *Aerococcus* spp. (*A. urinae* and *A. sanguinicola*) (2).

Lactobacilli are non-spore-forming, facultatively anaerobic or microaerophilic, non-nitrate-reducing, non-motile Gram-positive rods. These microorganisms are ubiquitous and widespread commensals in human and animal microbiota. In humans, they are usually considered non-pathogenic as they are common inhabitants of the oropharynx, the gastrointestinal tract, and the female genital tract. Like other lactic acid bacteria (e.g., pediococci, lactococci) and bifidobacteria, they are widely used as starter cultures in the food industry (for fermented milk products or sausages) or as probiotic preparations (3). Due to their fastidious growth under aerobic conditions and their resemblance to bacterial species of the resident microbiota, lactobacilli have frequently been overlooked or regarded as contaminants (4). Additionally, it has long been challenging to identify them to the species level using conventional phenotypic-based methods (5, 6). Although the advent of matrix-assisted laser desorption/ionization time-of-flight (MALDI-TOF) mass spectrometry has greatly improved their identification, molecular identification is still required in some cases (7). Nonetheless, they can occasionally cause invasive infections, such as bacteremia and endocarditis, which are the most frequent, followed by intra-abdominal and respiratory infections, UTIs, endophthalmitis, chorioamnionitis, and meningitis as well as various abscesses and wound infections (4–6, 8–14). The main species recovered from human infections, knowing that the genus *Lactobacillus* was extensively modified and several species have been reassigned to novel genus names, include *Lacticaseibacillus rhamnosus* (formerly *Lactobacillus rhamnosus*), *Lacticaseibacillus casei* (formerly *Lactobacillus casei*), and *Lactobacillus gasseri* followed by *Limosilactobacillus fermentum* (formerly *Lactobacillus fermentum*), *Lacticaseibacillus paracasei* (formerly *Lactobacillus paracasei*), *Lactobacillus jensenii*, *Lactobacillus acidophilus,* and *Lactiplantibacillus plantarum* (formerly *Lactobacillus plantarum*) (4–6, 8–11, 13–15). These infections are often associated with underlying conditions such as immunosuppression, neutropenia, cancer, diabetes, previous surgery or endoscopy, invasive procedures (central venous or urinary catheterization, intubation/ventilation, dental procedure), prosthetic material, renal failure, or prolonged ineffective antibiotic therapy (4, 5, 8).

*Lactobacillus delbrueckii* is the type species of the genus *Lactobacillus* and includes six different subspecies, namely *L. delbrueckii* subsp. *bulgaricus*, *L. delbrueckii* subsp. *delbrueckii*, *L. delbrueckii* subsp. *indicus*, *L. delbrueckii* subsp. *jakobsenii*, *L. delbrueckii* subsp. *lactis*, and *L. delbrueckii* subsp. *alloallosunkii* (formerly *L. delbrueckii* subsp. *sunkii*) (16–18). Among these subspecies, *bulgaricus* and *lactis* have been extensively studied as key microorganisms used in the food industry for producing fermented dairy products. By contrast, almost nothing is known about the other four subspecies. Interestingly, *L. delbrueckii* has rarely been reported as a cause of human infections, almost exclusively as a causative agent of UTIs, mainly in the elderly (10, 11, 13, 19–25). However, current knowledge (clinical relevance, antimicrobial susceptibility) about this species remains very limited. Additionally, it is not known whether human UTIs are due to specific subspecies.

The aim of this study was to (i) assess the clinical relevance of *L. delbrueckii* by analyzing the clinical and microbiological characteristics of 48 cases of probable UTIs, (ii) evaluate *in vitro* susceptibility to 15 antimicrobial agents and identify resistance genes using WGS, and (iii) determine the subspecies by comparing three genomic approaches.

## MATERIALS AND METHODS

### Isolates, media, growth conditions, and identification

We included 48 different clinical isolates of *L. delbrueckii* collected consecutively from urine samples of patients hospitalized at the Caen University Hospital between 2014 and 2016. From clinical specimens, isolates were grown on 5% horse blood agar plates (bioMérieux, Marcy-l’Etoile, France) and incubated anaerobically or under 5% CO_2_ at 35°C after 48 h of incubation with no difference in growth rates. Identification at the species level was performed using MALDI-TOF mass spectrometry (Microflex LT; Bruker Daltonics, Wissembourg, France; Biotyper software v3.0) after chemical extraction (ethanol, formic acid) in accordance with the manufacturer’s instructions.

### Antimicrobial susceptibility testing

MIC values were determined using the broth microdilution method as recommended by the 2022 EUCAST guidelines (http://www.eucast.org/ast_of_bacteria/). Mueller-Hinton broth with lysed horse blood (5%) and β-NAD (20 mg/L) (MHF; bioMérieux) was used, and plates were incubated for 48 h under 5% CO_2_ at 35°C. The following 15 antibiotics were tested: ampicillin, cefotaxime, imipenem, gentamicin, erythromycin, levofloxacin, tetracycline, tigecycline, vancomycin, teicoplanin, linezolid, daptomycin, cotrimoxazole, fosfomycin, nitrofurantoin, and metronidazole. Interpretation of results was based on CLSI breakpoints (https://em100.edaptivedocs.net/Login.aspx) for *Lactobacillus* spp. (ampicillin, imipenem, erythromycin, vancomycin, linezolid, and daptomycin). For daptomycin, the calcium content of MHF was adjusted to a final concentration of 50 mg/L. *Streptococcus pneumoniae* ATCC 49619 and *L. delbrueckii* subsp. *delbrueckii* CNRZ225 served as internal quality controls for each batch tested.

### Genome sequencing, assembly, and annotation

Genomic DNA from the 48 isolates was extracted using the Quick-DNA Fungal/Bacterial Miniprep Kit (Zymo Research, Irvine, CA, USA). DNA libraries were prepared using the NEBNext Ultra DNA Library Prep Kit for Illumina (New England Biolabs, Ipswich, MA, USA) and sequenced as paired-end reads (2 × 300 bp) on an Illumina MiSeq platform with the MiSeq Reagent Kit version 3. To ensure high-quality data, raw Illumina reads were trimmed using Trimmomatic (26) with a sliding window of four bases, discarding bases with a quality score below 20 and retaining reads with a minimum length of 50 bp. Genome assembly was performed using Unicycler v0.5.0 (27) using conservative mode and a minimum contig size of 1,000 bp. Genome annotation was conducted using Prokka (28), applying standard settings optimized for bacterial genomes. The assembled genomes have been deposited in GenBank under project accession PRJNA880510.

To investigate the presence of antimicrobial resistance genes, we used ResFinder 4.0 (29) with a minimum identity threshold of 90% and a minimum length threshold of 70%. Virulence genes were identified by aligning the genome sequences against the VFDB (30) database using BLAST with an E-value cutoff of 1e-5, ensuring the detection of genes with significant homology to known virulence factors.

### Comparative genomics and phylogenetics

For comparative analysis, 480 publicly available *L. delbrueckii* genomes were downloaded from the NCBI database (Table S1). Isolate discrimination was performed using multilocus sequence typing (MLST), average nucleotide identity (ANI), and phylogenomic approaches. *In silico,* MLST analysis was conducted using seven housekeeping genes (*fusA*, *gyrB*, *hsp60*, *ileS*, *pyrG*, *recA*, and *recG*) from 40 additional strains described by Tanigawa et al. (31) (Table S2). The concatenated sequences were aligned using MAFFT (32), and a minimum spanning tree was generated using GrapeTree (33) with the MStreeV2 algorithm to visualize the genetic relationships among the isolates.

Genomic similarity was assessed using Mash (34), a rapid tool for estimating pairwise genomic distances, with a sketch size of 1,000 and a k-mer size of 16. Mash outputs were processed using a custom Python script to generate an all-versus-all comparison of genomic distances and ANI values. We used an ANI cutoff below 98% for subspecies differentiation.

Pangenomic analysis was performed using Panaroo (35) with a 98% nucleotide identity cutoff in strict mode to reduce assembly artifacts, and core genome alignment was performed using MAFFT (32). To account for potential recombination events, we used Gubbins (36) to identify and mask regions affected by homologous recombination. Single nucleotide polymorphisms (SNPs) were extracted from the recombination-masked core genome alignment using SNP-sites (37). Pairwise SNP distances between isolates were calculated using PairSNP (https://github.com/gtonkinhill/pairsnp), using SNP alignment as input. A maximum-likelihood phylogenetic tree was constructed using IQ-TREE 2 (38), employing model selection and performing 1,000 ultrafast bootstrap replicates to ensure robust branch support. The resulting phylogenetic tree was midpoint-rooted and visualized using iTOL (39).

## RESULTS

### Clinical and microbiological characteristics

From 2014 to 2016, 48 clinical isolates of *L. delbrueckii* were recovered from the urine of 48 patients, including 37 (77%) from mid-stream samples and 11 from indwelling urinary catheters (Table 1). The median age of the patients was 84 years (range 54–96 years), with a large predominance of female patients (sex ratio M/F = 0.04) (Table 1). The majority of infections were community-acquired (*n* = 40, 83%). Only three (6%) patients had a predisposing urological disease, while 44 (92%) had at least one systemic underlying condition, including heart disease (*n* = 33, 69%), immunosuppression (*n* = 29, 60%), diabetes mellitus (*n* = 15, 31%), malignancy (*n* = 14, 29%), dementia (*n* = 12, 25%), and stroke (*n* = 6, 13%) (Table 1).

**TABLE 1:** Demographic, clinical, and microbiological characteristics of the 48 patients with urine specimens positive for *L. delbrueckii*

Characteristics	No. of patients (%)
Sex	
Male	2 (4)
Female	46 (96)
Age (years), median (range)	84 (54–96)
	
Origin	
Community-acquired	40 (83)
Hospital-acquired	8 (17)
Predisposing urologic disease	3 (6)
Indwelling urinary catheter	11 (23)
Systemic underlying condition(s)	
Heart disease	33 (69)
Immunosuppression	29 (60)
Mellitus diabetes	15 (31)
Malignancy	14 (29)
Dementia	12 (25)
Stroke	6 (13)
None	4 (8)
Diagnosis retained by the physician	
Infection Cystitis Pyelonephritis	21 (44) 11 (52) 10 (48)
Colonization/contamination	27 (6)
Antibiotic treatment for UTI	
Ceftriaxone	9 (19)
Amoxicillin-clavulanic acid	4 (8)
Amoxicillin	3 (6)
Ofloxacin	3 (6)
Ciprofloxacin	1 (2)
Piperacillin-tazobactam	1 (2)
	
Direct microscopic examination	
Leucocyturia (WBC count >10^4^/mL)	45 (94)
Gram staining positive for Gram-positive rods	43 (90)
Quantitative culture	
Significant *L. delbrueckii* count (≥10^5^ CFU/mL)	48 (100)
Concomitant uropathogen(s)^*a*^	27 (56)

^*a*^ *Escherichia coli* (*n* = 16), *Enterococcus faecalis* (*n* = 4), *Klebsiella pneumoniae* (*n* = 3), *Enterobacter cloacae* complex (*n* = 2), *Proteus mirabilis* (*n* = 1), *Citrobacter freundii* (*n* = 1).

Based on clinical and microbiological features, 21 (44%) patients were considered infected and initiated an antibiotic therapy, knowing that physicians were not aware of a potential uropathogenic role of *L. delbrueckii*. Of the 21 infected patients, 11 (52%) and 10 (48%) suffered from cystitis and pyelonephritis, respectively (Table 1). None of the patients developed severe complications such as endocarditis or urosepsis (data not shown). At the direct examination of the 48 urine specimens, 45 (94%) exhibited more than 10^4^ white blood cells (WBCs) per milliliter, while 43 (90%) showed Gram-positive rods (Table 1). All quantitative cultures had an *L. delbrueckii* bacterial count of ≥10^5^ CFU/mL, with a median of 10^6^ CFU/mL. *L. delbrueckii* was recovered in pure culture in 21 (44%) cases, while in mixed cultures, concomitant uropathogens were Gram-negative bacilli (incl. 16 *E. coli*, 3 *K*. *pneumoniae*, 2 *E. cloacae* complex, 1 *P*. *mirabilis,* and 1 *C*. *freundii*) or 4 *E. faecalis* (Table 1).

### Antimicrobial susceptibility profiles and treatments

All 48 *L*. *delbrueckii* isolates were fully susceptible to ampicillin, imipenem, erythromycin, vancomycin, linezolid, and daptomycin, while the majority exhibited low-level MIC_50_/MIC_90_ values for gentamicin (100%), cefotaxime (92%), tetracycline (90%), and cotrimoxazole (96%) (Table 2). MICs of teicoplanin and tigecycline were also low, suggesting a good *in vitro* activity of these antibiotics (Table 2). Levofloxacin appeared poorly active in most cases (MICs from 1 to 16 mg/L), while *L. delbrueckii* was likely intrinsically resistant to both fosfomycin and metronidazole, with MICs >256 mg/L for all tested strains (Table 2). Two isolates (LDEL-06 and LDEL-09) exhibited high MIC values for tetracycline (>16 mg/L), indicating acquired resistance compared to the putative wild-type population (MICs from 0.25 to 4 mg/L) (Table 2). Additionally, two putative resistant isolates (PR-7 and PR-9) were observed for cotrimoxazole (MIC = 8 mg/L), whereas wild-type strains showed MICs from 0.12 to 0.5 mg/L (Table 2).

**TABLE 2:** *In vitro* activity of 16 antimicrobial agents against 48 clinical isolates of *L. delbrueckii^b^*

Antibiotic	MIC (mg/L)			CLSI susceptibility breakpoint (mg/L)^*a*^	% of susceptible strains
	MIC_50_	MIC_90_	Range		
Ampicillin	0.12	0.25	0.03–0.5	8	100
Cefotaxime	0.5	1	0.03–2	–	–
Imipenem	0.06	0.12	0.01–0.5	0.5	100
Gentamicin	0.5	1	0.12–2	–	–
Erythromycin	0.06	0.12	0.01–0.5	0.5	100
Levofloxacin	4	16	1–16	-	-
Vancomycin	0.25	0.5	0.03–2	2	100
Teicoplanin	0.12	0.12	0.12–1	–	–
Linezolid	2	2	0.5–2	4	100
Tetracycline	1	2	0.25–>16	–	–
Tigecycline	0.25	0.5	0.12–0.5	–	–
Daptomycin	0.12	0.5	0.06–0.5	4	100
Cotrimoxazole	0.12	0.25	0.12–8	–	–
Nitrofurantoin	16	32	8–32	–	–
Fosfomycin	>256	>256	>256	–	–
Metronidazole	>256	>256	>256	–	–

^*a*^ CLSI breakpoints for *Lactobacillus* spp. were used for ampicillin, imipenem, erythromycin, vancomycin, linezolid, and daptomycin (CLSI M45-ED3:2016 Methods for antimicrobial dilution and disk susceptibility testing of infrequently isolated or fastidious bacteria, third edition).

^*b*^ "-" means not available.

### Genomic analysis

All 48 isolates were unambiguously identified at the species level using MALDI-TOF mass spectrometry; however, phenotypically distinguishing the different subspecies was not possible. Consequently, the genomes of all 48 isolates were sequenced and analyzed. The mean genome size was 1.99 ± 0.06 Mb with a G+C content of 49.9% ± 0.1%, and the mean number of coding sequences was 1,911 ± 49 (Table 3). The core genome comprised 1,123 orthologous gene families, while the pan-genome was open, consisting of 5,616 orthologous gene families.

**TABLE 3:** Genomic data of the 48 *L*. *delbrueckii* clinical isolates^*a*^

Strain ID	*L. delbrueckii* subspecies	Assembly size (Mb)	GC%	No. of CDS	Resistance genes	No. of contigs	N50	L50	Coverage (X)	GenBank accession number
LDEL02	*allosunkii*	2.06	49.77	1,934	–	48	74,998	8	255	JAYSIN000000000
LDEL04	*allosunkii*	2.05	49.77	1,927	–	49	73,394	7	332	JAYSIM000000000
LDEL-06	*allosunkii*	2.08	49.84	1,985	*tet*(W)	72	54,390	12	166	GCA_026891255.1
LDEL-09	*allosunkii*	2.11	49.77	2,004	*tet*(W)	64	65,483	11	130	GCA_026891265.1
LDEL10	*allosunkii*	2.10	49.66	2,014	–	73	53,111	13	243	JAYSIL000000000
LDEL11	*allosunkii*	1.95	50.17	1,867	–	66	68,921	10	273	JAYSIK000000000
LDEL13	*lactis*	1.94	49.57	1,927	–	96	42,923	13	280	JAYSIJ000000000
LDEL14	*allosunkii*	2.02	49.95	1,933	–	73	58,626	14	254	JAYSII000000000
LDEL19	*lactis*	1.97	49.76	1,947	–	81	46,214	16	316	JAYSIH000000000
LDEL21	*allosunkii*	1.96	49.92	1,882	–	87	40,366	14	243	JAYSIG000000000
LDEL22	*allosunkii*	2.01	49.92	1,925	–	76	46,593	10	310	JAYSIF000000000
LDEL24	*allosunkii*	1.96	50.18	1,864	–	44	102,800	7	322	JAYSIE000000000
LDEL25	*allosunkii*	2.05	49.76	1,926	–	47	74,998	7	287	JAYSID000000000
LDEL26	*allosunkii*	2.02	49.94	1,888	–	50	68,962	8	302	JAYSIC000000000
LDEL28	*lactis*	1.95	49.74	1,937	–	86	39,415	17	276	JAYSIB000000000
LDEL29	*allosunkii*	2.05	49.77	1,936	–	54	68,962	9	329	JAYSIA000000000
LDEL32	*allosunkii*	2.06	49.62	1,989	–	109	28,910	23	239	JAYSHZ000000000
PR01	*allosunkii*	2.00	49.93	1,928	–	74	47,985	14	268	JAYSHY000000000
PR02	*allosunkii*	1.84	49.91	1,788	–	125	23,936	24	305	JAYSHX000000000
PR03	*allosunkii*	1.94	50.03	1,863	–	113	24,343	24	312	JAYSHW000000000
PR04	*lactis*	1.87	50.02	1,858	–	102	32,919	19	347	JAYSHV000000000
PR05	*allosunkii*	2.05	49.78	1,929	–	52	74,998	9	382	JAYSHU000000000
PR06	*lactis*	1.86	49.98	1,818	–	78	41,365	13	341	JAYSHT000000000
PR-7	*allosunkii*	2.06	49.76	1,939	–	50	73,394	8	130	GCA_026891475.1
PR08	*allosunkii*	2.00	49.74	1,937	–	117	28,753	21	329	JAYSHS000000000
PR-9	*lactis*	1.95	49.70	1,934	–	84	46,215	14	102	GCA_026891305.1
PR10	*allosunkii*	2.05	49.78	1,931	–	61	65,416	12	283	JAYSHR000000000
PR11	*lactis*	1.95	49.75	1,942	–	96	33,599	21	310	JAYSHQ000000000
PR12	*allosunkii*	2.02	50.07	1,909	–	69	52,964	11	250	JAYSHP000000000
PR15	*allosunkii*	1.94	49.85	1,860	–	95	46,718	15	309	JAYSHO000000000
PR16	*allosunkii*	2.01	49.87	1,912	–	56	67,249	8	316	JAYSHN000000000
PR17	*allosunkii*	1.98	50.02	1,873	–	55	73,383	7	270	JAYSHM000000000
PR18	*allosunkii*	2.05	49.78	1,936	–	66	64,800	10	288	JAYSHL000000000
PR19	*allosunkii*	2.06	49.76	1,933	–	51	73,393	8	247	JAYSHK000000000
PR20	*allosunkii*	1.96	50.17	1,869	–	53	90,809	9	244	JAYSHJ000000000
PR21	*allosunkii*	2.00	49.84	1,913	–	75	54,621	12	197	JAYSHI000000000
PR22	*allosunkii*	1.93	50.03	1,843	–	86	35,400	15	380	JAYSHH000000000
PR23	*allosunkii*	1.92	49.94	1,844	–	101	27,694	20	346	JAYSHG000000000
PR24	*allosunkii*	2.07	49.91	1,972	–	57	84,947	8	293	JAYSHF000000000
PR25	*lactis*	1.98	49.72	1,957	–	76	41,571	16	333	JAYSHE000000000
PR26	*allosunkii*	1.98	49.84	1,900	–	112	27,934	22	285	JAYSHD000000000
PR27	*allosunkii*	2.01	49.95	1,896	–	54	73,394	8	276	JAYSHC000000000
PR29	*allosunkii*	2.04	49.78	1,925	–	57	68,962	8	155	JAYSHB000000000
PR30	*allosunkii*	1.93	50.14	1,832	–	122	26,713	23	205	JAYSHA000000000
PR31	*allosunkii*	2.05	49.77	1,933	–	52	67,160	8	275	JAYSGZ000000000
PR32	*allosunkii*	1.99	49.90	1,912	–	66	57,585	10	256	JAYSGY000000000
PR33	*allosunkii*	1.97	49.89	1,884	–	114	24,314	22	317	JAYSGX000000000
PR34	*allosunkii*	1.93	50.03	1,833	–	56	59,273	11	223	JAYSGW000000000

^*a*^ "-" means not found.

**Fig 1:**
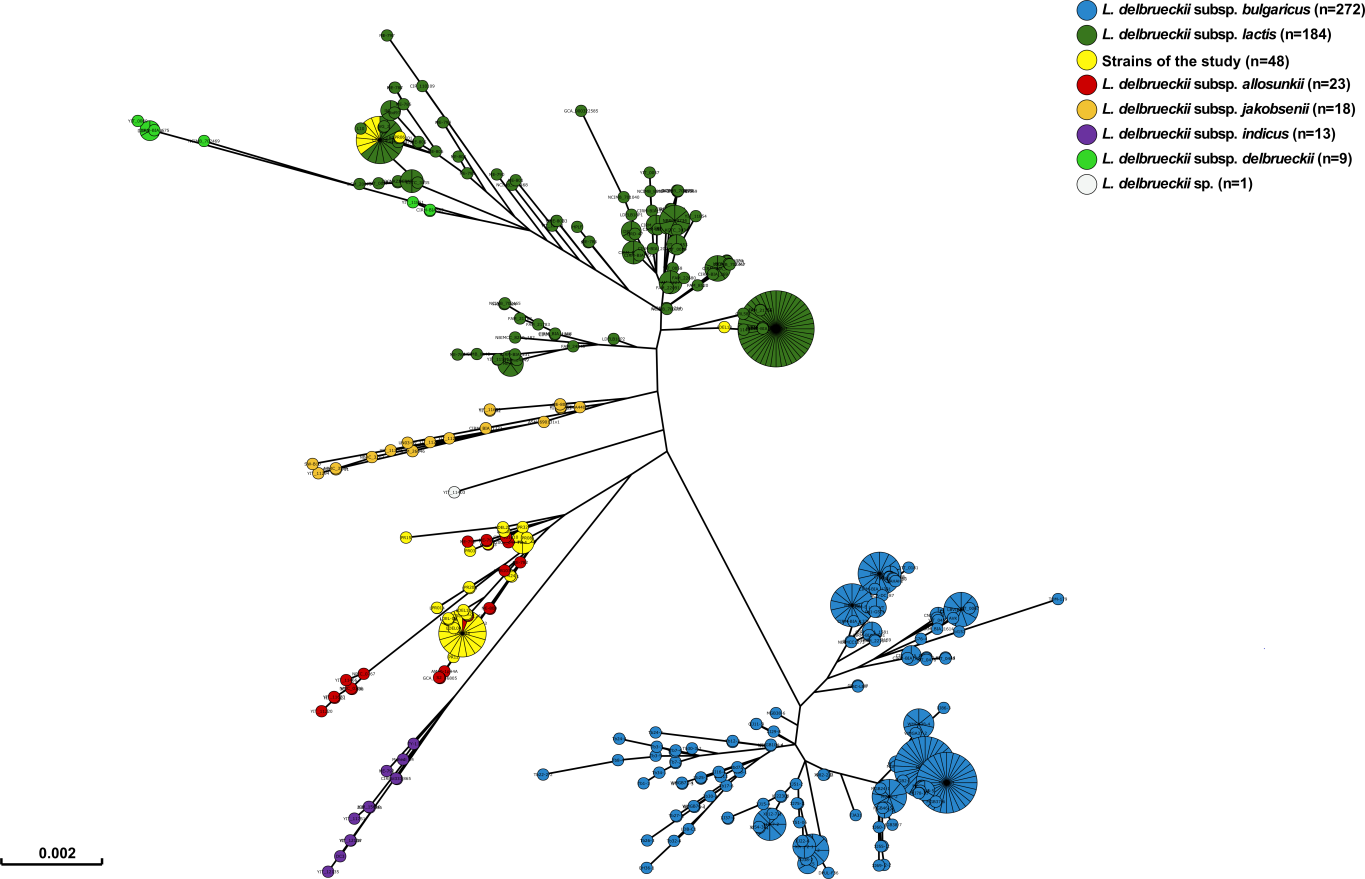
Minimum spanning tree based on MLST data from 568 *L*. *delbrueckii* isolates. The collection of isolates includes 480 isolates from NCBI, 40 strains described by Tanigawa et al. (Table S2) (31), and the 48 clinical isolates of the study. The seven housekeeping genes (*fusA*, *gyrB*, *hsp60*, *ileS*, *pyrG*, *recA,* and *recG*) were aligned against all genomes, and the tree was built from concatenated sequences using GrapeTree (https://github.com/achtman-lab/GrapeTree). Clinical isolates of the study are colored in yellow.

**Fig 2:**
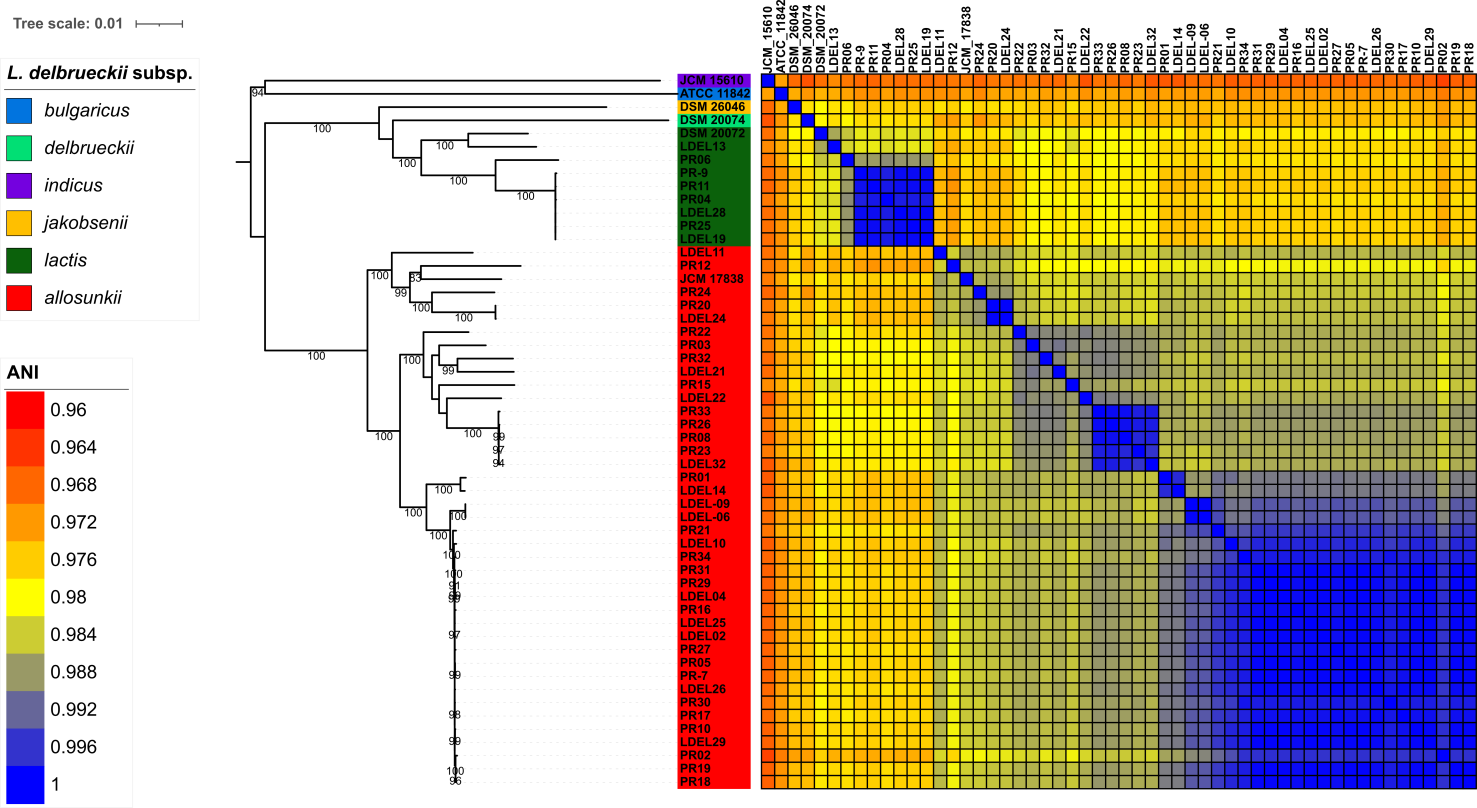
ANI analysis of the 48 isolates compared with *L. delbrueckii* reference subspecies genomes. The maximum-likelihood phylogenetic tree was constructed using a core genome SNP-based approach with IQ-TREE 2 (TVM+F+ASC+G4 best-fit model) and 1,000 ultrafast bootstrap iterations (http://www.iqtree.org/). The tree was visualized using iTOL (https://itol.embl.de). The reference strains included are *L. delbrueckii* subsp. *allosunkii* JCM 17838, *L. delbrueckii* subsp. *lactis* DSM 20072, *L. delbrueckii* subsp. *bulgaricus* ATCC 11842, *L. delbrueckii* subsp. *delbrueckii* DSM 20074, *L. delbrueckii* subsp. *indicus* JCM 15610, and *L. delbrueckii* subsp. *jakobsenii* DSM 26046.

**Fig 3:**
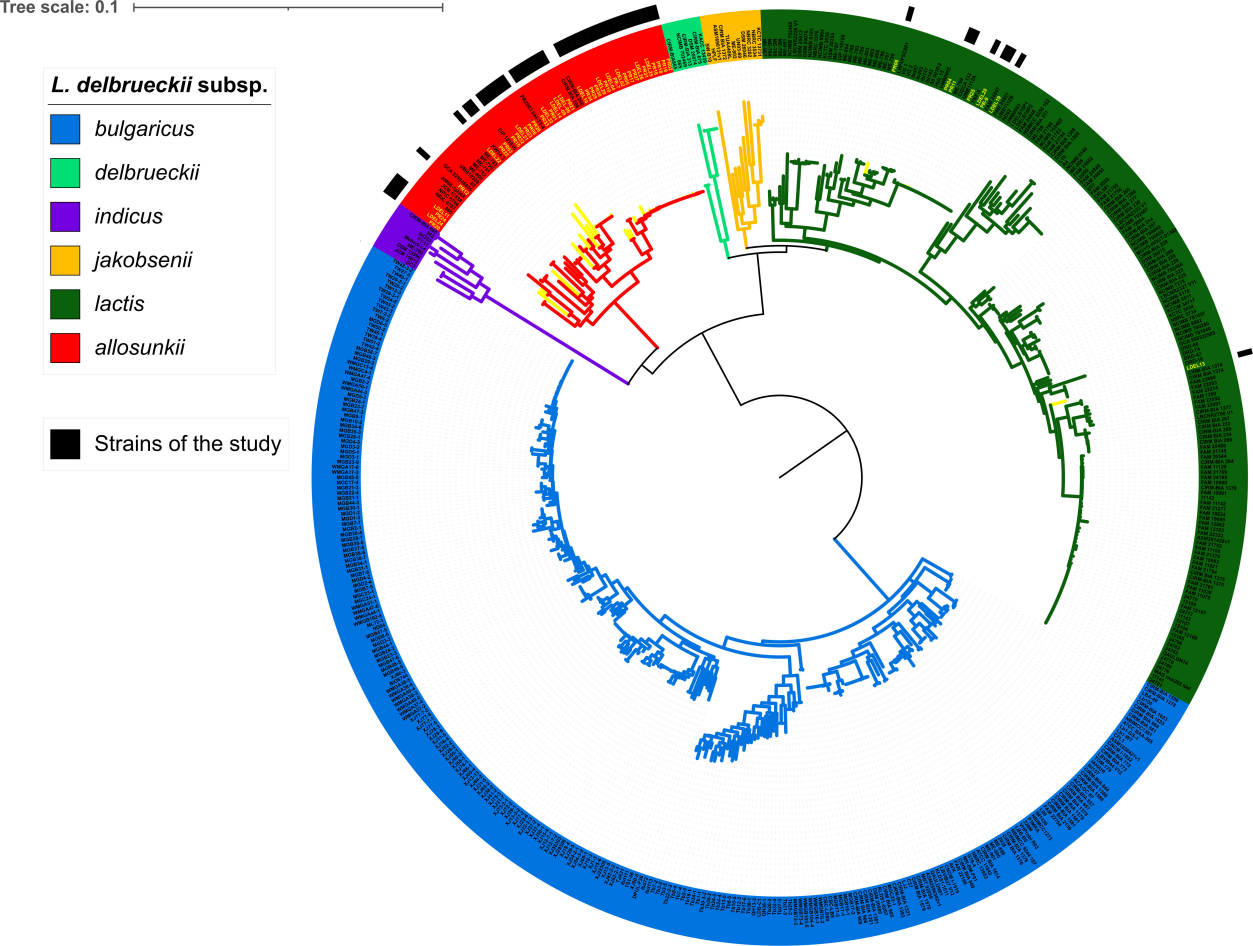
Core genome SNP-based maximum-likelihood tree of 528 *L*. *delbrueckii* isolates. This data set comprises 480 isolates from NCBI and 48 clinical isolates from this study. The tree was constructed using IQ-TREE 2 (GTR+F+ASC+G4 best-fit model) with 1,000 ultrafast bootstrap iterations (http://www.iqtree.org/), based on 132,691 polymorphic sites within the core genome. Visualization was performed using iTOL (https://itol.embl.de). Clinical isolates from this study are indicated by black rectangles, and their names are highlighted in yellow.

**Fig 4:**
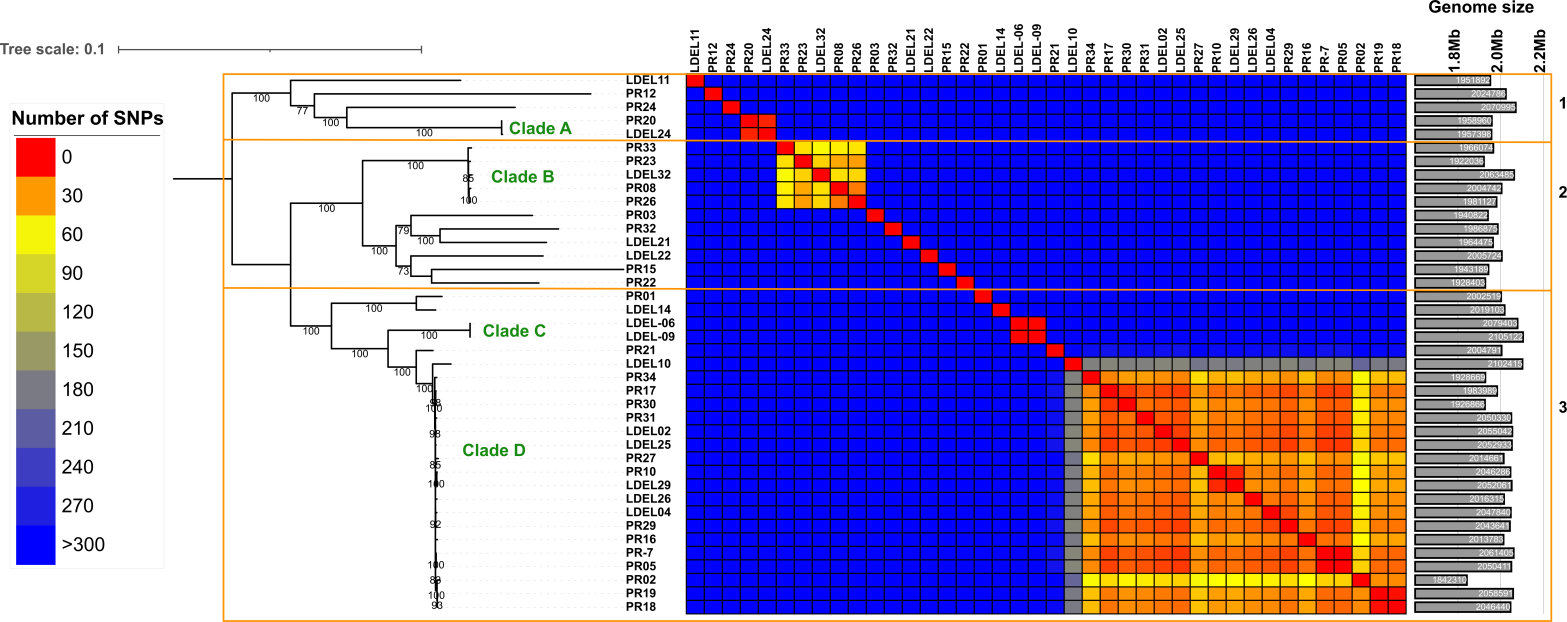
Maximum-likelihood phylogenetic tree based on the alignment of 10,137 recombination-masked SNPs derived from 1,207 core gene sequences from the 40 *L*. *delbrueckii* subsp. *allosunkii* clinical isolates. A heatmap is provided to illustrate SNP distances among the isolates. The three primary lineages (1–3) and four clades (**A–D**) are indicated.

Subspecies identification was initially performed using MLST, revealing that most *L. delbrueckii* clinical isolates belonged to the subspecies *allosunkii* (*n* = 40; 83%), followed by L. *delbrueckii* subsp. *lactis* (*n* = 8; 17%) (Fig. 1). This distribution was further confirmed through ANI analysis. The ANI values for the 40 strains identified as *allosunkii*, compared to the *L. delbrueckii* subsp. *allosunkii* reference strain JCM 17838 ranged from 98.12% to 98.74%. Similarly, ANI values for the eight strains identified as *lactis*, relative to the *L. delbrueckii* subsp. *lactis* reference strain DSM 20072, ranged from 98.18% to 98.68% (Fig. 2; Fig. S1; Table S3). The subspecies distribution was also supported by a core genome SNP-based phylogenomic approach (Fig. 3), which included an analysis of 480 additional *L. delbrueckii genomes*. To assess genomic diversity within *L. delbrueckii* subsp. *allosunkii* (*n* = 40), a phylogenetic tree was constructed based on the alignment of 10,187 recombination-masked SNPs from 1,207 core gene sequences. This analysis revealed three major lineages, each comprising distinct sublineages (Fig. 4). The average genome sizes for lineages 1, 2, and 3 were 1.88, 1.94, and 1.97 Mb, respectively (Fig. 4). Notably, four clades of clonally-related strains were identified: clade A (2 isolates, 7 SNPs), clade B (5 isolates, 24 to 55 SNPs), clade C (2 isolates, 1 SNP), and clade D (18 isolates, 3 to 58 SNPs) (Fig. 4; Table S4). These findings highlight the genomic diversity and structure within the *L. delbrueckii* subsp. *allosunkii* population.

Both tetracycline-resistant clinical isolates (LDEL-06 and LDEL-09) belonged to the subspecies *allosunkii* and harbored the *tet*(W) gene on their chromosomes, which encodes a ribosomal protection protein that confers resistance by preventing tetracycline from binding to the ribosome (Table S5). By analysis of sequences from NCBI, we also identified a *tet*(W) gene in the strain *L. delbreuckii* subsp. *indicus* ME-792 (Table S5). For cotrimoxazole, no acquired resistance genes or mutations were detected. Finally, no obvious virulence genes were found (Table S6).

## DISCUSSION

Similar to the aforementioned emerging Gram-positive uropathogens, lactobacilli are likely underreported in the medical literature as they are typically regarded as commensals or contaminants, leading to the absence of an actual prevalence. For information, the prevalence over the period of these “minor” uropathogens represented less than 2% of bacterial species recovered from urine specimens, incl. *A. urinae* (1%), *A. schaalii* (0.4%), *L. delbrueckii* (0.2%), and *A. sanguinicola* (0.2%) (data not shown). The most commonly reported infections due to lactobacilli are bacteremia and infective endocarditis, with *L. rhamnosus*, *L. paracasei*, *L. plantarum*, *L. fermentum*, *L. casei*, *Lactobacillus crispatus*, *Lactobacillus johnsonii*, *L. jensenii*, *L. acidophilus*, *Ligilactobacillus salivarius* (formerly *Lactobacillus salivarius*), *L. gasseri*, and *Latilactobacillus curvatus* (formerly *Lactobacillus curvatus*) being the most frequent species (4–6, 8–10, 14, 16). Studies on virulence have mainly focused on *L. rhamnosus* and *L. paracasei* (12). These species possess potential virulence factors, such as enzymes that break down human glycoproteins and proteins that bind to extracellular proteins such as fibronectin, fibrinogen, and collagen, facilitating early-stage colonization and adherence. Some strains also have the ability to aggregate human platelets and produce biofilm (13).

Recent metagenomic analyses revealed that *L. delbrueckii* inhabits the intestines of humans and animals (40). In healthy individuals, this species represents the fourth most prevalent species among lactobacilli after *Ligilactobacillus ruminis* (formerly *Lactobacillus ruminis*), *L. casei*, and *L. gasseri* in the gut microbiota (40). UTIs caused by lactobacilli are very uncommon, with the vast majority attributed to *L. delbrueckii* (only 14 reported cases) (19–25). Among these 14 patients, there was a predominance of women (12/14, 86%), and most patients were elderly, with a median age of 82 years (range: 49–94 years). The two male patients suffered from acute bacterial prostatitis (23, 25). For all patients, urine dipsticks were negative for nitrite detection, consistent with the fact that lactobacilli do not reduce nitrate to nitrite (16). As reported in our study, urinalysis showed in all cases pyuria, positive direct examination with Gram-positive rods, and significant culture (>10^5^ CFU/mL), usually monobacterial (19–25). The main putative risk factors reported here and in previous case reports are old age, diabetes mellitus, hypothyroidism, dementia, benign prostate hyperplasia, and urolithiasis (19, 21–25). Invasive infections caused by *L. delbrueckii* are much less frequently reported, with only a few cases of bacteremia described (8–10, 24), and no invasive infections were observed in our case series.

Only two subspecies were involved in UTIs in our study. *L. delbrueckii* subsp. *allosunkii* was first isolated from sunki, a traditional Japanese non-salted pickle, while *L. delbrueckii* subsp. *lactis* is heavily used for the production of Parmesan- and Emmental-type cheeses (15, 17, 18). Out of the 14 cases of UTIs caused by *L. delbrueckii* reported in the literature, the subspecies has been mentioned in only three cases (one *lactis*, one *delbrueckii,* and one *allosunkii*). This is surprising since the identification in these three clinical cases was only obtained by MALDI-TOF mass spectrometry (22–24), which is not a reliable method for identification at the subspecies level.

Genomic analysis of our collection revealed a mean genome size of 1.99 ± 0.06 Mb, a G+C content of 49.9% ± 0.1%, and a core genome of 1,123 genes, similar to what has been recently described in a comparative analysis of 31 *L*. *delbrueckii* genomes, which reported a genome size of around 1.93 ± 0.16 Mb, a G+C content of 49.8% ± 0.4%, and a core genome of 1,069 orthologous gene families (17).

According to the type of sugar fermentation pathway, lactobacilli are separated into three major metabolic groups that form distinct phylogenetic clades: obligately homofermentative, facultatively heterofermentative, and obligately heterofermentative (12, 15). Interestingly, homofermentative lactobacilli (e.g., *L. acidophilus*, *Lactobacillus amylovorus*, *L. crispatus*, *L. delbrueckii*, *L. gasseri*, *Lactobacillus helveticus*, *L. jensenii*, *L. johnsonii*) are susceptible to vancomycin and teicoplanin, whereas heterofermentative lactobacilli (e.g., *Levilactobacillus brevis* [formerly *Lactobacillus brevis*], *L. casei*, *L. curvatus*, *L. fermentum*, *L. paracasei*, *Lactiplantibacillus pentosus* [formerly *Lactobacillus pentosus*], *L. plantarum*, *Limosilactobacillus reuteri* [formerly *Lactobacillus reuteri*], *L. rhamnosus*, *Latilactobacillus sakei* [formerly *Lactobacillus sakei*], *L. salivarius*, *Lacticaseibacillus zeae* [formerly *Lactobacillus zeae*]) are inherently highly resistant to these antibiotics (4–6, 9, 41–43). As anticipated for *L. delbrueckii*, all isolates in our study demonstrated susceptibility to vancomycin (MIC_50_/_90_: 0.25/0.5 mg/L) and teicoplanin (MIC_50_/_90_: 0.12/0.12 mg/L). Lactobacilli are also intrinsically susceptible to aminopenicillins, imipenem, erythromycin, tetracycline, and linezolid, whereas the *in vitro* activity of cephalosporins, gentamicin, fluoroquinolones, cotrimoxazole, and nitrofurantoin appears more variable depending on the species (4–6, 9, 41, 43, 44). Among newer antibiotics, tigecycline and linezolid typically exhibit good activity, whereas daptomycin shows variable efficacy (42). Finally, the intrinsic resistance to metronidazole of lactobacilli is well documented (41, 42).

Most data on *L. delbrueckii* originates from food isolates, particularly the subspecies *bulgaricus* and *lactis*. Though few human isolates have been tested for antimicrobial susceptibility, they seem to share similar profiles with food isolates, showing susceptibility to ampicillin, imipenem, gentamicin, erythromycin, tetracycline, vancomycin, linezolid, and daptomycin (9, 11, 19, 21, 24, 25, 42, 45). Fluoroquinolones exhibit variable activity, with ciprofloxacin being less active than levofloxacin/moxifloxacin, and some evidence of acquired resistance exists (9, 11, 19). Notably, high-level resistance to fosfomycin has been reported (20, 22, 23).

Tetracycline and erythromycin resistance genes are the most common in lactobacilli, with *tet*(M) and *erm*(B) being the most widespread and often genetically linked (42, 46). Few resistance genes have been found in some food isolates of *L. delbrueckii* subsp. *bulgaricus* such as *tet*(M), *aph(3’)-III*, and *ant(6)* (42, 45). Together with our results, this suggests that acquired resistance in *L. delbrueckii* is uncommon.

UTIs caused by *L. delbrueckii* have been successfully treated with β-lactams (aminopenicillins, piperacillin-tazobactam, cefixime) or clarithromycin, indicating that the recommended treatment for *L. delbrueckii*-related infections should rely on β-lactams, particularly aminopenicillins (20–22, 24). It is important to note that recurrent UTIs caused by *L. delbrueckii* were often linked to treatment failures with fluoroquinolones, fosfomycin, or cefuroxime (19, 21–23, 25).

Despite the retrospective observational monocentric design, this study shows the potential uropathogenic role of *L. delbrueckii* (especially the subspecies *allosunkii*) in UTIs among elderly patients and those with predisposing conditions. We also demonstrate that these clinical isolates are generally susceptible to many antibiotics, with only a few intrinsic and acquired resistances. Based on *in vitro* data, the antibiotic treatment of these infections should rely on aminopenicillins rather than fosfomycin or fluoroquinolones.

## SUPPLEMENTAL MATERIAL

Figure S1 jcm.02072-24-s0001.pdf Core genome SNP-based maximum likelihood tree of 528 *L. delbrueckii isolates*.

Table S1 jcm.02072-24-s0002.xlsx List of publicly-available *L. delbrueckii* genomes (n = 480) downloaded from the NCBI database (https://www.ncbi.nlm.nih.gov/) for comparative genomics and phylogenetics.

Table S2 jcm.02072-24-s0003.xlsx List of additional strains described by Tanigawa et al. used for MLST analysis.

Table S3 jcm.02072-24-s0004.xlsx Results of the average nucleotide identity (ANI) analysis for the 528 *L. delbrueckii* genomes (480 from NCBI + 48 clinical isolates of the study).

Table S4 jcm.02072-24-s0005.xlsx Results of the single nucleotide polymorphism (SMP) pangenomic analysis for the 40 *L. delbrueckii* subsp. *allosunkii* clinical isolates.

Table S5 jcm.02072-24-s0006.xlsx List of antimicrobial resistance genes detected in the collection of 528 *L. delbrueckii* strains by using ResFinder 4.0 (minimum identity threshold of 90%; minimum length threshold of 70%).

Table S6 jcm.02072-24-s0007.xlsx List of putative virulence genes detected in the collection of 48 *L. delbrueckii* clinical isolates using the VFDB database.

ASM does not own the copyrights to Supplemental Material that may be linked to, or accessed through, an article. The authors have granted ASM a non-exclusive, world-wide license to publish the Supplemental Material files. Please contact the corresponding author directly for reuse.
