# Supplementary figures and images for: Lactobacillus delbrueckii subsp. allosunkii and lactis as emerging human uropathogens in elderly patients
Source: J Clin Microbiol. 2025 Apr 23;63(5):e02072-24. doi: 10.1128/jcm.02072-24 (PMC12077209; doi:10.1128/jcm.02072-24)

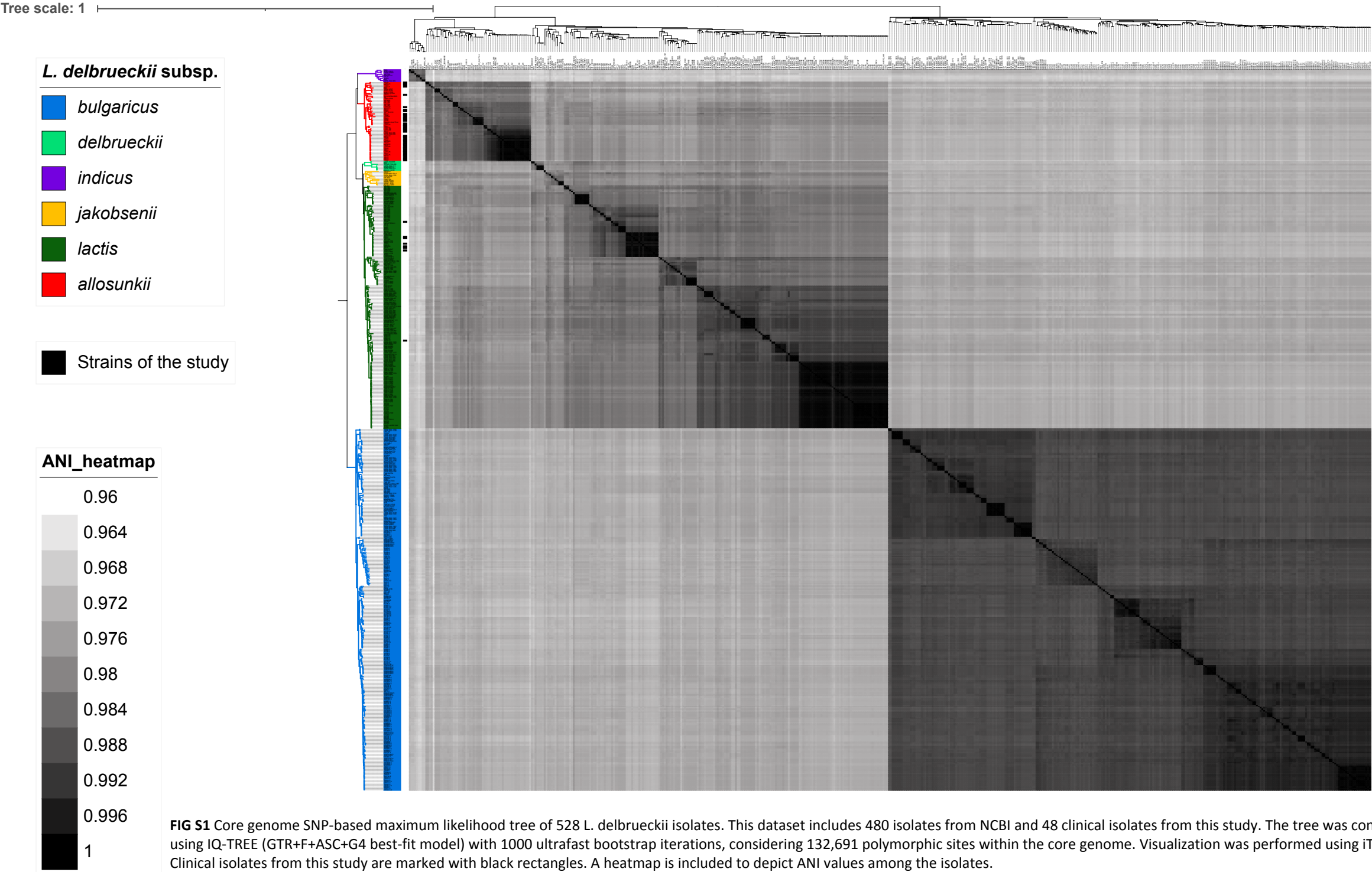

Supplement: Figure S1 — Core genome SNP-based maximum likelihood tree of 528 L. delbrueckii isolates. [file jcm.02072-24-s0001.pdf]
